# The prediction of two-dimensional PbN: opened bandgap in heterostructure with CdO

**DOI:** 10.3389/fchem.2024.1382850

**Published:** 2024-04-18

**Authors:** Zhang Cheng, Yuelei Wang, Ruxin Zheng, Weihua Mu

**Affiliations:** ^1^ Department of Automotive and Mechanical Engineering, Anhui Communications Vocational & Technical College, Hefei, China; ^2^ Faculty of Mechanical and Electrical Engineering, Hainan Vocational University of Science and Technology, Haikou, China; ^3^ School of Mechanical Engineering, Southeast University, Nanjing, China; ^4^ Wenzhou Institute, University of Chinese Academy of Sciences, Wenzhou, China

**Keywords:** two-dimensional materials, PBN, mechanical properties, heterostructure, applications

## Abstract

The development of two-dimensional (2D) materials has received wide attention as a generation of optoelectronics, thermoelectric, and other applications. In this study, a novel 2D material, PbN, is proposed as an elemental method using the prototype of a recent reported nitride (*J. Phys. Chem. C* 2023, 127, 43, 21,006–21014). Based on first-principle calculations, the PbN monolayer is investigated as stable at 900 K, and the isotropic mechanical behavior is addressed by the Young’s modulus and Poisson’s ratio at 67.4 N m^–1^ and 0.15, respectively. The PbN monolayer also presents excellent catalytic performance with Gibbs free energy of 0.41 eV. Zero bandgap is found for the PbN monolayer, and it can be opened at about 0.128 eV by forming a heterostructure with CdO. Furthermore, the PbN/CdO is constructed by Van der Waals interaction, while the apparent potential drop and charge transfer are investigated at the interface. The PbN/CdO heterostructure also possesses excellent light absorption properties. The results provide theoretical guidance for the design of layered functional materials.

## 1 Introduction

After the discovery of graphene (Geim and Novoselov, 2009), two-dimensional (2D) materials have received much investigation due to its remarkable electronic (Ghosh et al., 2015; Ambrosi et al., 2017), optical (Mogulkoc et al., 2016), mechanical (Sun et al., 2017), and catalytic (Lathe et al., 2023) properties and a wide range of applications. For example, black phosphorus possesses a puckered structure, which can be prepared by electrochemical method under a field effect transistor. The thickness of black phosphorus can reach several nanometers, and the highest carrier mobility can be as high as 10^4^ cm^2^·v^−1^·s^–1^ (Xiao et al., 2015). At room temperature, black phosphorus more than 7.5 nm thick presents excellent transistor performance, and the leakage current modulation is of 10^5^ order (Li et al., 2014; Jia et al., 2015; Carvalho et al., 2016; Clark et al., 2018). As the allotrope of black phosphorus, blue phosphorus is hexagonal in plane and has a bandgap of 2.77 eV (Ren et al., 2019a). The hopping parameters of the TB Hamiltonian of blue phosphorus are extracted by density functional theory (DFT), and it has been found that the energy band of blue phosphorus can be tuned by the applied electric field (Ding and Wang, 2015; Li et al., 2015; Liu et al., 2015; Zhu et al., 2016; Xiong et al., 2017; Yang et al., 2017; Safari et al., 2019). The arsenene also shows a honeycomb structure and has an indirect bandgap in both bending and folding states (Kamal and Ezawa, 2015), which can be further tuned by applying strain. It is worth noting that 1% external strain can transform the puckered arsenene into a direct semiconductor (Kamal and Ezawa, 2015; Zhang et al., 2015; Wang et al., 2017; Xu et al., 2017; Wang et al., 2018; Zhang et al., 2018). All this demonstrates that 2D materials have favorable prospects in optoelectronics and thermoelectric nanodevices.

In order to further expand the application of these 2D materials and discover more unusual mechanical, optical, and electronic properties, many strategies have been explored to predict and develop new 2D materials (Miao and Sun, 2022). Wu et al. (2016) have predicted a family of titanium silicide (Ti_2_Si, TiSi_2,_ and TiSi_4_) monolayers through the calculation of DFT, in which Ti_2_Si is a ferromagnetic metal, and the magnetic moment is obtained as 1.37 *μ*_B_/cell. TiSi_2_ has been proven to be an ideal catalyst with excellent hydrogen evolution reaction performances. Importantly, TiSi_4_ can be used as a powerful 2D phonon-mediated superconductor; its transition temperature is calculated as 5.8 K, and the transition temperature in particular can be increased to 11.7 K by applying a certain strain. Luo et al. (2011) used the global optimization method to predict 2D boron carbon. The calculation results demonstrate that BC compounds exhibit metal properties. In addition, BC_3_ shows semiconductor properties, and the most stable BC structure possesses high thermal stability even above 2000 K. Based on evolutionary search and first principles, Sun and Schwingenschlögl (2020) predicted an anisotropic Janus structure material B_2_P_6_, which is a semiconductor with an indirect bandgap of 2.09 eV. Interestingly, the Janus structure can induce an inherent electric field, which significantly inhibits the recombination of optical source carriers. It is also proven that B_2_P_6_ can be used as a promising photocatalyst for water splitting, with an excellent solar-to-hydrogen efficiency of 28.2%. Lu et al. (2018) predicted a 2D material CaP_3_, which has a direct bandgap of 1.15 eV and ultrahigh electron mobility of about 2.0 × 10^4^ cm^2^·v^−1^·s^–1^. Jing et al. (2017) proposed a GeP_3_ semiconductor with an indirect bandgap of 0.55 eV. It is notable that GeP_3_ can transform the indirect bandgap into the direct bandgap under the condition of biaxial strain. GeP_3_ also has remarkable light absorption ability and can be widely used in the field of optoelectronics.

In this study, we propose a new 2D material named PbN by the elemental method with the prototype of the recent reported XN monolayers (Ren et al., 2023a). Using first-principle calculations, the stability and structural parameters are addressed. Then, the mechanical, electronic, and catalytic performances are investigated. The Van der Waals based on the PbN and CdO monolayers is constructed with an opened tiny bandgap, which also possesses excellent optical properties. The results show that the PbN/CdO van der Waals heterostructure is promising as an optical nano-sensor.

## 2 Computational methods

All simulations in this work were carried out by the first-principles method, which is employed by density functional theory (DFT) (Grest et al., 1981; Capelle, 2006). The calculation instrument is the Vienna *Ab initio* simulation package (VASP) (Van de Walle and Martin, 1989), which is embedded with the projector-augmented wave method (Kresse and Furthmüller, 1996a; Kresse and Joubert, 1999; Grimme et al., 2010). The Perdew–Burke–Ernzerhof functional was considered by utilizing the generalized gradient approximation means (Kresse and Furthmüller, 1996b; Perdew et al., 1996; Grimme, 2006). The phonon spectra of the system under study were explored by the density functional perturbation theory (DFPT), which is obtained by PHONOPY code (Togo et al., 2008; Togo and Tanaka, 2015). In calculations of the band structure of the studied heterostructure, the Heyd–Scuseria–Ernzerhof hybrid functional (Heyd et al., 2005) was explored to calculate a more precise bandgap, and the DFT-D3 method was considered for the heterostructure to address the weak dispersion forces proposed by Grimme et al. (2010). To simulate the Gibbs free energy of the studied system, the DFT-D3 method of Grimme was explored to consider the Van der Waals interactions in all calculations, and the dipole-corrected functional was also adopted (Van de Walle and Martin, 1989). The energy cut-off was selected as 550 eV, and the Monkhorst–Pack *k*-point grid is 17 × 17 × 1 in the first Brillouin zone. The vacuum height was adopted by 25 Å, which can effectively prevent forces between adjacent layers. The convergence for force was used by 0.01 eV Å^−1^, and the energy of the calculated system was controlled in 0.01 meV.

## 3 Results and discussion

**FIGURE 1:**
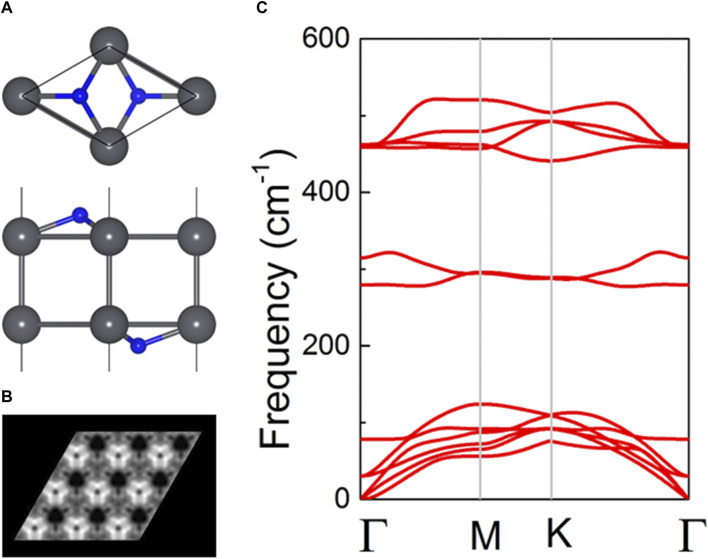
**(A)** Atomic structure of the PbN monolayer; gray and blue balls are Pb and N atoms, respectively. **(B)** Simulated STM images of the PbN crystal structures under the bias of −2 V. **(C)** Calculated phonon spectrum of the PbN monolayer.

The predicted atomic structure of the PbN monolayer is shown in Figure 1A, constructed by the elemental method (Ren et al., 2022a). This crystal structure of the PbN monolayer is honeycombed with a space group of *P3m1*, inspired by the SiN monolayer prototype (Ren et al., 2023a). It is worth noting that there is also a possibility that the PbN monolayer possesses other phase structures, but we just focused on the crystal structures of the predicted NSi monolayer prototype. The cohesive energy *E*) of the PbN monolayer is calculated by *E* = [*E*_N2_ + *E*_Pb bulk_ − *E*_PbN_/2], where *E*_PbN_, *E*_N2_, and *E*_Pb bulk_ are the total energy of the PbN system, N_2_, and Pb bulk, respectively. After the optimization of the PbN monolayer, the obtained cohesive energy of the PbN monolayer is 6.41 eV/atom, which is comparable to early synthesized 2D materials like MoS_2_ (about 5.02 eV/atom) (Sun and Schwingenschlögl, 2021) and SiN (about 5.56 eV/atom) (Ren et al., 2023a). Importantly, the cohesive energies of the monolayered PbN show an energetic stability, which presents a feasibility in experimental preparation. The lattice constant obtained for the monolayered PbN is calculated as 3.653 Å, which is larger than that of the MoS_2_ monolayer (3.180 Å) (Ren et al., 2019b). The simulated scanning tunneling microscopy (STM) appearance of the monolayered PbN with 3 × 3 supercell is expressed by Figure 1B, which is beneficial for identifying the structure of the PbN monolayer in future experiments. Furthermore, the phonon dispersion of the PbN monolayer is calculated to verify dynamic stabilities (Togo et al., 2008; Togo and Tanaka, 2015), suggested as Figure 1C. It can be seen that no imaginary frequency is obtained in the phonon dispersion, explaining a dynamic stability of the PbN monolayer. It is worth noting that the highest frequency of the PbN monolayer is comparable with that of SnN (Ren et al., 2023a).

**FIGURE 2:**
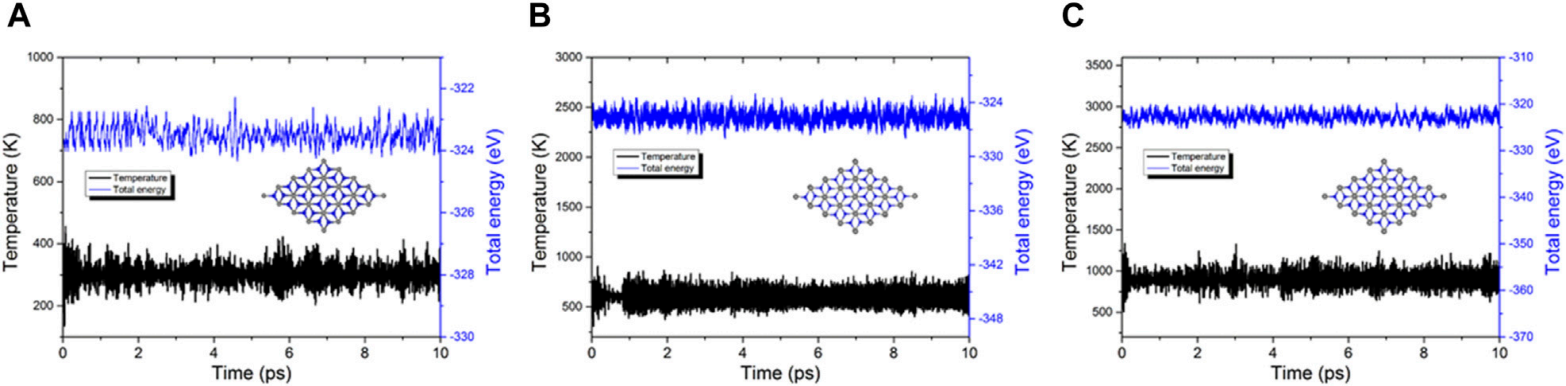
Temperature and energy in AIMD simulations of the PbN monolayer at **(A)** 300 K, **(B)** 600 K, and **(C)** 900 K; the insets are AIMD snapshots of the PbN, the gray and blue balls are Pb and N atoms, respectively.

Investigating the stability of the PbN monolayer requires not only consideration of the cohesive and phonon spectrum but also the thermal property that requires further study. Thus, AIMD calculations are conducted for further exploration. The PbN system is constructed by a 5 × 5 × 1 supercell with 100 atoms in the simulations to prevent the lattice translational constraints (Li et al., 2023). The heat bath scheme is selected by the Nosé–Hoover method (Nosé, 1984). After completing the calculations, the relaxed structure of the monolayered PbN is still undamaged at 300 K, 600 K, and 900 K (Figures 2A–C, respectively) for 10 ps. The obtained temperature and total energy are monitored as stable in the AIMD calculation, suggesting a robust thermal stability of the predicted PbN monolayer.

**FIGURE 3:**
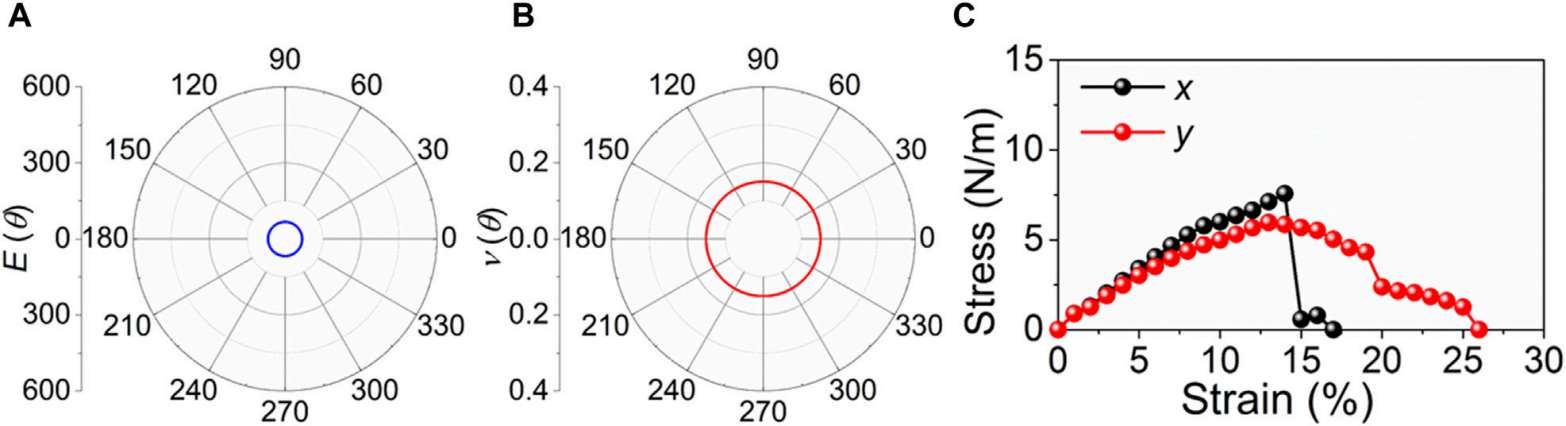
Calculated **(A)** Young’s moduli, **(B)** Poisson’s ratios, and **(C)** strain–stress relationships along the *x* and *y* directions of the PbN monolayer.

We next investigated the mechanical performance of the PbN monolayer by calculating Young’s modulus *E*) and Poisson’s ratio *v*), defined as:

$$E\left(\theta \right)=\frac{C_{11}C_{22}-C_{12}^{2}}{C_{11}\sin^{4}\theta +C_{22}\cos^{4}\theta +\left(\frac{C_{11}C_{22}-C_{12}^{2}}{C_{66}}-2C_{12}\right)\cos^{2}\theta \sin^{2}\theta },$$ (1)

$$v\left(\theta \right)=-\frac{\left(C_{11}+C_{22}-\frac{C_{11}C_{22}-C_{12}^{2}}{C_{66}}\right)\cos^{2}\theta \sin^{2}\theta -C_{12}\left(\cos^{4}\theta +\sin^{4}\theta \right)}{C_{11}\sin^{4}\theta +C_{22}\cos^{4}\theta +\left(\frac{C_{11}C_{22}-C_{12}^{2}}{C_{66}}-2C_{12}\right)\cos^{2}\theta \sin^{2}\theta },$$ (2)

where *θ* is the angle beginning the *x*-direction. Young’s modulus and Poisson’s ratio of the PbN monolayer are expressed by Figures 3A,B, respectively. One can see that Young’s modulus and Poisson’s ratio of the PbN monolayer do not present directional dependence, suggesting isotropic mechanical property (Ren et al., 2023a). The calculated Young’s modulus and Poisson’s ratio of the PbN monolayer are 67.4 N m^–1^ and 0.15, respectively. Such a small Young’s modulus of the PbN monolayer forced the nanocomponents, while it is still higher than that of ZnO (47.8 N m^–1^) (Peng et al., 2013). The obtained Poisson’s ratio of the PbN monolayer is also smaller than that of MX_2_Y_4_ monolayers (0.264–0.327) (Ren et al., 2023b) and larger than that of the CN monolayer (about 0.12) (Ren et al., 2023a). The strain effect of the PbN monolayer is also calculated along the *x* and *y* directions. Even if the PbN monolayer presents an apparently isotropic Young’s modulus and Poisson’s ratio, the fracture property is different along the *x* and *y* directions. Shown as Figure 3C, the PbN monolayer exhibits significant fracture stress about 7 N m^–1^ at 14% along the *x* direction, while it only has yield limit in the *y* direction of about 6 N m^–1^ at 13%. It is worth noting that in the small strain range, the slopes in both the *x* and *y* directions are the same, which also confirms the equality of the Young’s modulus of the PbN monolayer (Sun et al., 2020).

**FIGURE 4:**
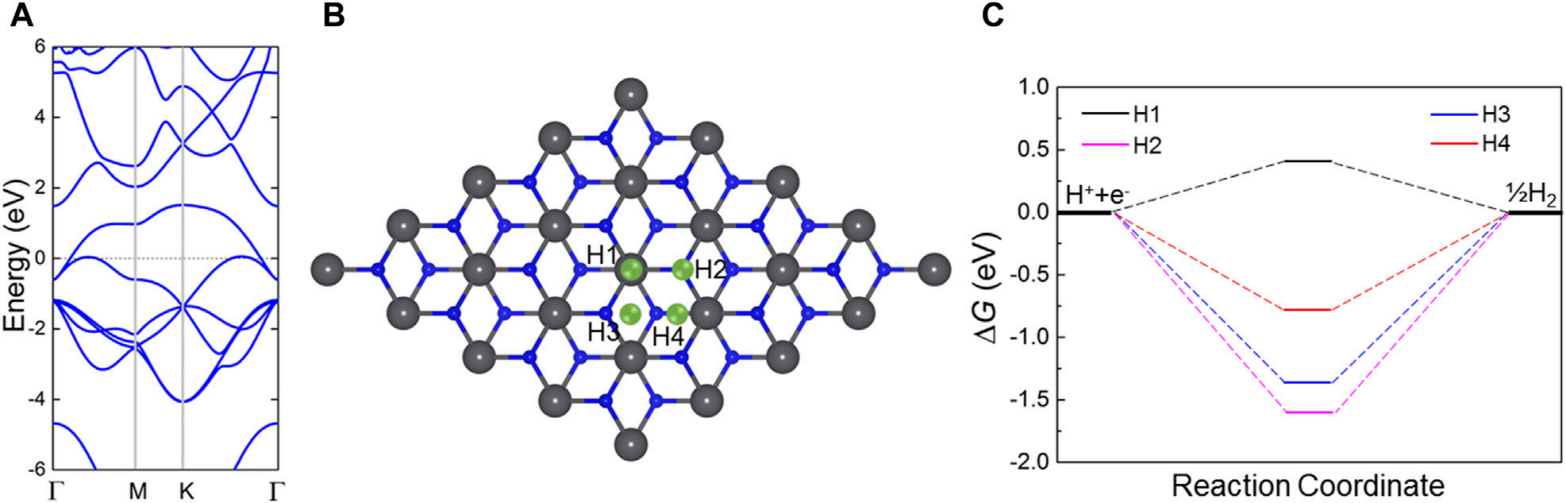
**(A)** Calculated band structure of PbN monolayer; Fermi level is 0. **(B)** Representative adsorption sites in hydrogen evolution reaction of the PbN monolayer. **(C)** Calculated Gibbs free energies of the PbN monolayer in the hydrogen evolution reaction.

The band structure of the PbN monolayer is then calculated by HSE06 calculations (Figure 4A). On can see that the Fermi level passes through the band energy with a zero-bandgap characteristic. The hydrogen evolution reaction performance of the PbN monolayer is further explored considering the active adsorption point marked by the cyan balls in Figure 4B. Obviously, four different representative active adsorption points are addressed by the highly symmetrical structure. The Gibbs free energy changes (Δ*G*_H*_) of the PbN monolayer are decided at standard conditions using

$$\Delta G_{H^{*}}=\Delta E+\Delta E_{\text{zpe}}+T\Delta S,$$ (3)

where the energy of the H adsorbed PbN monolayer is Δ*E*. The difference of the zero-point energy is expressed by Δ*E*_zpe_. The difference in the entropy by the hydrogen evolution reaction is Δ*S*. In this study, *T* is considered 298.15 K in the simulations. The active site is demonstrated by “*”. Thus, the hydrogen evolution reaction process is finished by two reactions:

$$*+H^{+}+e^{-}\;\to \;H^{*},$$ (4)

$$H^{*}+H^{+}+e^{-}\;\to \;H_{2}+*.$$ (5)

The most stable HER adsorption configuration is decided via binding energy (*E*_b_), which is calculated by *E*_b_ = *E*_system_ – *E*_pure_ – *E*_H_, where *E*_system_, *E*_pure_, and *E*_H_ represent the energy of the adsorbed PbN system, pure PbN monolayer, and single H atom, respectively. The lower binding energy implies a more stable structure; thus, the most stable HER adsorption site is obtained by the system with lowest binding energy in the H1 site. After the calculations, the Gibbs free energy of the PbN monolayer with these active sites is obtained as Figure 4C. The PbN monolayer possesses excellent catalytic performance, with Gibbs free energy of 0.41 eV at the H1 active site, which is even more favorable than that of the biphenylene network (about 2.93 eV) (Ren et al., 2022b) and graphene (about 1.41 eV) (Luo et al., 2021).

**FIGURE 5:**
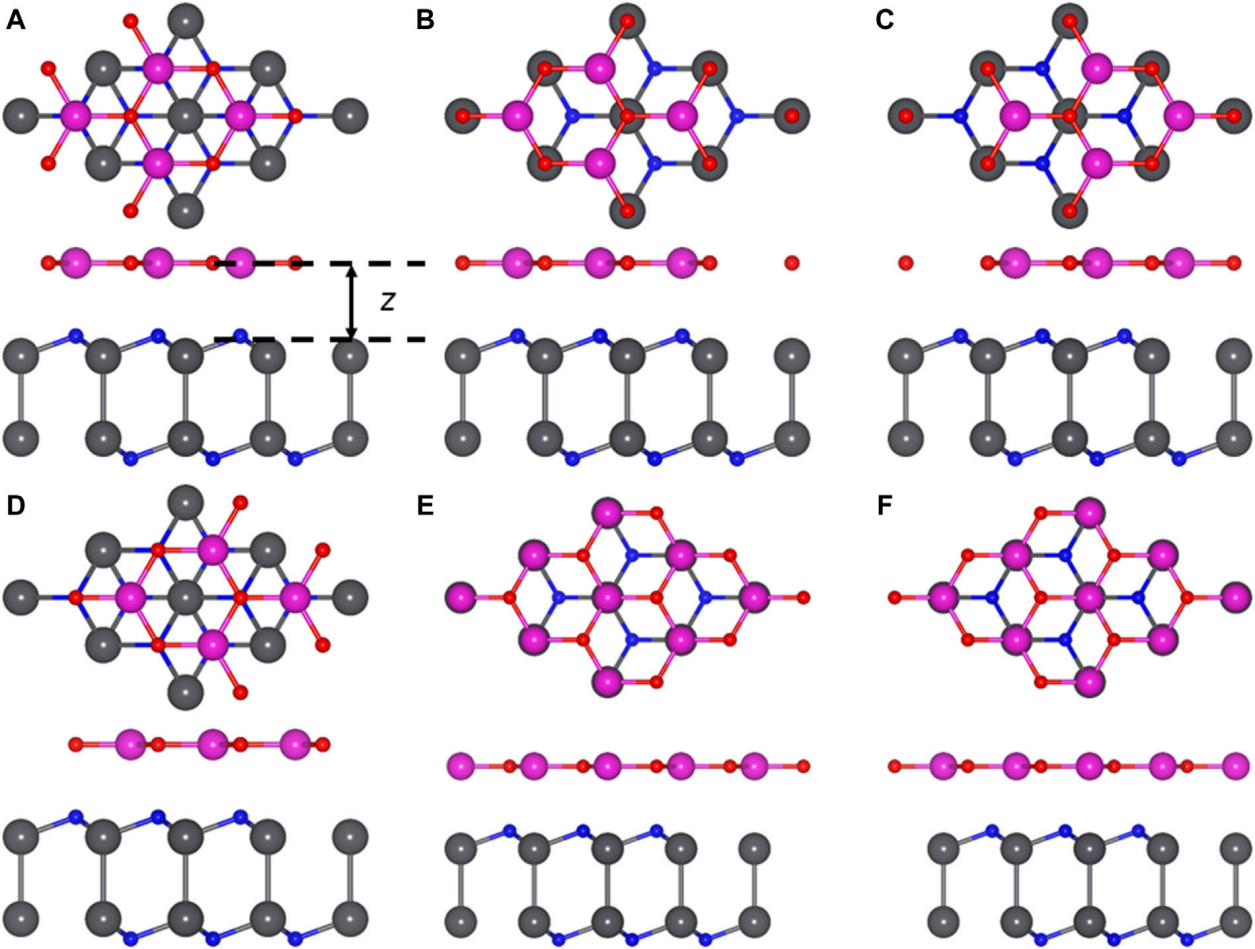
Stacked PbN/CdO heterostructure with representative structures **(A)** NC-1, **(B)** NC-2, **(C)** NC-3, **(D)** NC-4, **(E)** NC-5, **(F)** NC-6. Gray, blue, red, and purple balls are Pb, N, O, and Cd atoms, respectively.

Considering that the lattice constant of the monolayered PbN as 3.653 Å is comparable with that of the CdO monolayer (3.684 Å) (Ren et al., 2021), the Van der Waals (vdWs) heterostructure formed by the PbN and CdO monolayers is feasible. Thus, the lattice mismatch is only about 0.8% in the PbN/CdO heterostructure, while six different representative stacking structures of the PbN/CdO heterostructure should be considered by high symmetry (Figure 5)—NC-1, NC-2, NC-3, NC-4, NC-5, and NC-6. For NC-1, the PbN/CdO heterostructure is constructed by the O and Cd atoms on the top of the lower and upper N atoms, respectively. Then, the NC-2 PbN/CdO heterostructure is formed by the O and Cd atoms on top of the Nb and upper N atoms, respectively. In NC-3 PbN/CdO heterostructure, the O and Cd atoms are located on top of the Nb and lower N atoms, respectively. The O and Cd atoms are on top of the upper N and the lower N, and the NC-4 PbN/CdO heterostructure is obtained. For the NC-5 PbN/CdO heterostructure, the O and Cd atoms are fixed on top of the upper N and Nb atoms, respectively. Furthermore, the NC-6 PbN/CdO heterostructure can be constructed by locating the O and Cd atoms on top of the lower N and Nb atoms, respectively.

To determine the most stable PbN/CdO heterostructure with these six different configurations, the binding energy *E*) of the PbN/CdO heterostructure is considered by *E* = *E*_PbN/CdO_ − *E*_PbN_ − *E*_CdO_, where *E*_PbN/CdO_, *E*_PbN_, and *E*_CdO_ are the energy of the PbN/CdO system, pure PbN, and CdO monolayers, respectively. The obtained binding energy of the PbN/CdO heterostructure is −30.11 meV/Å^2^ for NC-2 configuration, which is lower than that of graphene (about −18 meV/Å^2^) (Chen et al., 2013). Thus, the PbN/CdO heterostructure is constructed by vdWs interactions. In addition, the distance of the interface and bond length of these different stacking configurations of the optimized PbN/CdO heterostructure and pure PbN, CdO monolayers are calculated in Table 1. The obtained interfacial high of the NC-2 PbN/CdO heterostructure is 2.414 Å, which is smaller than that of the CdO/HfS_2_ vdW heterostrutcure (about 2.86 Å) (Zhang et al., 2022a). The following calculations are based on this structure.

**TABLE 1:** Obtained binding energy (*E*, meV/Å^2^), distance of interface (*z*, Å), and bond length (*L*, Å) of the PbN/CdO heterostructure with different stacking styles and pure PbN, CdO.

	*E*	*z*	*L*_N–Pb_	*L*_Cd–O_
PbN			2.246	
CdO				2.117
NC-1	−29.57	2.442	2.273	2.122
NC -2	−30.11	2.414	2.258	2.114
NC -3	−27.09	2.489	2.258	2.109
NC -4	−27.14	3.111	2.267	2.119
AO-5	−23.63	3.315	2.263	2.114
NC -6	−22.44	2.837	2.681	2.112

**FIGURE 6:**
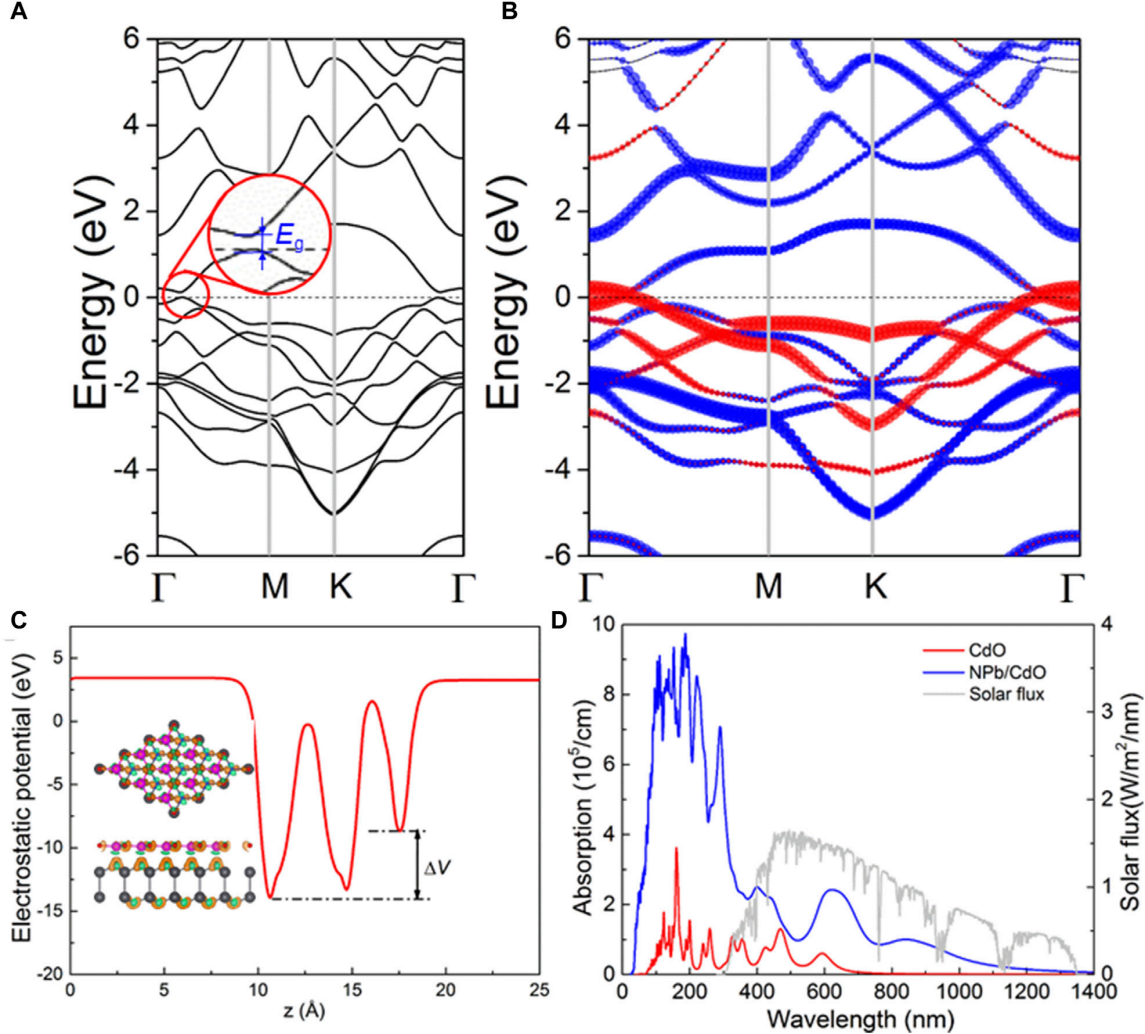
Calculated **(A)** band structure and **(B)** projected band structure of PbN/CdO vdW heterostructure; red and blue marks are contributed PbN and CdO monolayers, respectively. **(C)** Potential drop of PbN/CdO vdW heterostructure; inset is the isosurface lever for charge difference in the PbN/CdO vdW heterostructure, the orange and blue ones represent the gain and loss of electrons, respectively. **(D)** HSE06 obtained optical absorption coefficient of the PbN/CdO vdW heterostructure.

The band energy of the PbN/CdO vdWs heterostructure is calculated in Figure 6A; interestingly, a very small bandgap (about 0.128 eV) is found. Furthermore, the projected band structure of the PbN/CdO vdWs heterostructure is calculated in Figure 6B using the HSE06 functional. It can be seen that the slight bandgap results from the PbN layer, meaning that the bandgap of the PbN monolayer can be opened by forming a vdWs heterostructure with the CdO monolayer. Thus, the PbN/CdO vdWs heterostructure is a potential candidate for use as a sensor in nano-devices. In Figure 6C, the potential difference across the interface of the PbN/CdO vdWs heterostructure is obtained as 5.323 eV, which is larger than that of other reported heterostructures, such as MoTe_2_/PtS_2_ (4.41–4.67 eV) (Ren et al., 2022c), MoTe_2_/PtS_2_ (4.67 eV) (Zhang et al., 2022b), and CdO/HfS_2_ (5.23 eV) (Zhang et al., 2022a). Furthermore, the charge density difference (Δ*ρ*) of the PbN/CdO vdWs heterostructure was also investigated, demonstrated by Δ*ρ* = *ρ*_PbN/CdO_ − *ρ*_PbN_ − *ρ*_CdO_, where *ρ*_PbN/CdO_, *ρ*_PbN_ and *ρ*_CdO_ being the total charge of the PbN/CdO system, and the pure PbN and CdO monolayers, respectively. The results show that CdO obtains considerable electrons (about 13.619) by Bader-charge analysis (Sanville et al., 2007). This also explains the larger potential drop across the interface of the PbN/CdO vdWs heterostructure in Figure 6C. Such desirable potential drop is also of great assistance for carrier migration between the interface of the PbN/CdO vdWs heterostructure. The tiny bandgap in the PbN/CdO vdWs heterostructure compared to the wide bandgap in the CdO monolayer (about 2.073 eV) can also enhance the light absorption properties, expressed as

$$\alpha \left(\omega \right)=\frac{\sqrt{2}\omega }{c}{\left\{{\left[\varepsilon_{1}^{2}\left(\omega \right)+\varepsilon_{2}^{2}\left(\omega \right)\right]}^{1/2}-\varepsilon_{1}\left(\omega \right)\right\}}^{1/2},$$ (6)

where *ω* is used as the angular frequency, *α* is the absorption coefficient, and the speed of light is demonstrated by *c*. $\varepsilon_{1}\left(\omega \right)$ and $\varepsilon_{2}\left(\omega \right)$ explain the real and imaginary characteristics in the dielectric constant, respectively. The complex dielectric function is *ε*(*ω*) = *ε*_1_(*ω*) + *iε*_2_(*ω*), and the real part *ε*_1_ can be obtained from *ε*_2_ through the Kramers–Kronig relation. *ε*_1_(*ω*) and *ε*_2_(*ω*) can be calculated by Zhang et al. (2008):

$$\varepsilon_{2}\left(q\to O_{\hat{u}},\hbar \omega \right)=\frac{2e^{2}\pi }{\Omega \varepsilon_{0}}\sum_{k,v,c}|\langle \Psi_{k}^{c}|{\left.\hat{u}\cdot r\left|\Psi_{k}^{v}\right.\rangle \right|}^{2}\times \delta \left(E_{k}^{c}-E_{k}^{v}-E\right),$$ (7)

where $\Psi_{k}$ , $E_{k},$ and $\hat{u}$ are the wave function, energy, and unit vector of the electric field of the incident light, respectively. The superscripts (*v* and *c*) in $\Psi_{k}$ and $E_{k}$ label the conduction and valence bands, respectively. Shown as Figure 6D, the PbN/CdO vdWs heterostructure possesses excellent optical performance to the CdO; the light absorption peaks are as high as 2.51 × 10^5^ cm^−1^ and 2.4 × 10^5^ cm^−1^ at wavelengths of 400 nm and 597 nm, respectively, in the visible light range. The obtained light absorption capacity of the PbN/CdO vdWs heterostructure is also higher than that of other 2D heterostructures, such as ZnO/BSe (8.72 × 10^4^ cm^−1^) (Ren et al., 2020) and MoSSe/Mg(OH)_2_ (1.66 × 10^5^ cm^−1^) (Lou et al., 2021). Thus, the PbN/CdO vdWs heterostructure is a promising application as an optical sensor.

## 4 Conclusion

A novel 2D nitride, PbN, is predicted in this investigation. Structure and stability is systematically studied with the PbN monolayer presenting dynamic and thermal stability at 900 K. The Young’s modulus and Poisson’s ratio of the PbN monolayer are calculated as 67.4 N m^–1^ and 0.15, respectively. The excellent catalytic performance of the PbN monolayer is also obtained with the Gibbs free energy of 0.41 eV. The PbN monolayer is formed as a heterostructure with a CdO monolayer, which is constructed by vdW forces. The PbN/CdO can open a tiny bandgap for the PbN (about 0.128 eV), and the predominant light absorption performances is also addressed. The results show that the PbN monolayer has potential as an optical sensor.

## Data Availability

The original contributions presented in the study are included in the article/Supplementary Material; further inquiries can be directed to the corresponding author.
